# Cardiac Biomarker Complete Response in AL Amyloidosis: Characteristics, Cardiac Recovery, and Survival of 63 Patients

**DOI:** 10.1016/j.jaccao.2026.01.004

**Published:** 2026-04-21

**Authors:** Eli Muchtar, Susan Geyer, Angela Dispenzieri, Martha Grogan, Taxiarchis V. Kourelis, Francis K. Buadi, Prashant Kapoor, David Dingli, Wilson Gonsalves, Nelson Leung, Giampaolo Merlini, Julie Rosenthal, Barry Boilson, Melissa Lyle, Janell Grazzini Frantz, Suzanne R. Hayman, Martha Q. Lacy, Mustaqeem Siddiqui, Joselle Cook, Nadine Abdallah, Moritz Binder, Saurabh Zanwar, Yi Lin, Rahma Warsame, Robert A. Kyle, S. Vincent Rajkumar, Shaji K. Kumar, Morie A. Gertz

**Affiliations:** a Division of Hematology, Mayo Clinic, Rochester, Minnesota, USA; b Division of Clinical Trials and Biostatistics, Mayo Clinic, Rochester, Minnesota, USA; c Division of Cardiovascular Disease, Mayo Clinic, Rochester, Minnesota, USA; d Division of Nephrology and Hypertension, Mayo Clinic, Rochester, Minnesota, USA; e Department of Molecular Medicine, University of Pavia, Pavia, Italy; f Amyloidosis Research and Treatment Center, Fondazione IRCCS Policlinico San Matteo, Pavia, Italy; g Department of Cardiovascular Medicine, Mayo Clinic, Phoenix, Arizona, USA; h Division of Advanced Heart Failure and Transplant, Department of Transplantation, Mayo Clinic, Jacksonville, Florida, USA

**Keywords:** AL amyloidosis, biomarkers, cardiac biomarkers, clone-directed therapy, complete response, echocardiography, outcomes, survival, treatment

## Abstract

**Background:**

Cardiac biomarker complete response (CR) is a new concept in amyloid light chain (AL) cardiac amyloidosis (CA).

**Objectives:**

The aim of this study was to characterize patients with AL CA who achieved cardiac biomarker CR, including clinical presentation, treatment, cardiac recovery, and survival.

**Methods:**

This single-center retrospective cohort study included patients diagnosed with AL CA between 2004 and 2023 who met cardiac biomarker response eligibility (baseline N-terminal pro–B-type natriuretic peptide [NT-proBNP] >650 pg/mL or B-type natriuretic peptide >150 pg/mL). Among these, patients who achieved cardiac biomarker CR, defined as NT-proBNP ≤350 pg/mL or B-type natriuretic peptide ≤80 pg/mL sustained for at least 12 months, were identified. The median follow-up duration was 9.5 years.

**Results:**

Sixty-three patients achieved cardiac biomarker CR (4.7% of evaluable cases [63 of 1,342]). This proportion increased in the second period compared with the first (6.4% [38 of 591] vs 3.3% [25 of 751]; *P* < 0.001). The median age was 57 years (Q1-Q3: 49-66 years), and 63.5% were men. The median baseline difference between involved and uninvolved free light chains was 429 mg/L (Q1-Q3: 143-708 mg/L), the median pretreatment NT-proBNP concentration was 1,977 pg/mL, and 57.1% of patients were in cardiac stage II. Hematologic CR preceded cardiac biomarker CR in 76% of patients. The median time to cardiac biomarker CR was 20.6 months. At cardiac biomarker CR, the median NT-proBNP concentration was 265 pg/mL (86.9% reduction from baseline). Echocardiographic parameters improved by the time of cardiac biomarker CR but did not fully normalize in all patients. Cardiac progression occurred in 14% of patients, and 44.4% required subsequent clone-directed therapy. Eight patients died, 2 of non–AL CA–related causes. Survival was comparable with that of a matched general U.S. population (*P* = 0.35).

**Conclusions:**

Cardiac biomarker CR represents the deepest level of cardiac biochemical recovery in AL amyloidosis and is associated with survival similar to the general population. Despite biochemical recovery, structural cardiac abnormalities may persist, underscoring the importance of early diagnosis and timely therapy.

Amyloid light chain (AL) amyloidosis is a plasma cell disorder marked by extracellular deposition of misfolded light chains as amyloid fibrils, most commonly affecting the heart and kidneys.^1^ Cardiac involvement typically presents as restrictive cardiomyopathy with systemic hypoperfusion and heart failure symptoms.^2^ Conduction system abnormalities are also common, predisposing patients to atrial and ventricular arrhythmias and conduction delays. Cardiac disease severity is the primary driver of prognosis.^3^ Median survival for patients with AL amyloidosis and cardiac involvement has historically been short, often measured in months rather than years, especially in those with advanced heart failure symptoms.^4^ Cardiac staging, based on N-terminal pro–B-type natriuretic peptide (NT-proBNP) and troponin levels, stratifies prognosis, with higher levels indicating poorer outcomes.^5^, ^6^, ^7^

Achieving a cardiac biomarker complete response (CR), defined as NT-proBNP ≤350 pg/mL or B-type natriuretic peptide (BNP) ≤80 pg/mL, is a strong prognostic marker. Patients reaching cardiac biomarker CR may have 5-year survival rates approaching 93%, similar to matched general populations.^8^ In this study, we characterized clinical presentation, treatment, response kinetics, cardiac recovery, and survival among patients who achieved cardiac biomarker CR. The trajectory and clinical implications of cardiac biomarker CR in AL amyloidosis are summarized in the Central Illustration.

**Central Illustration:**
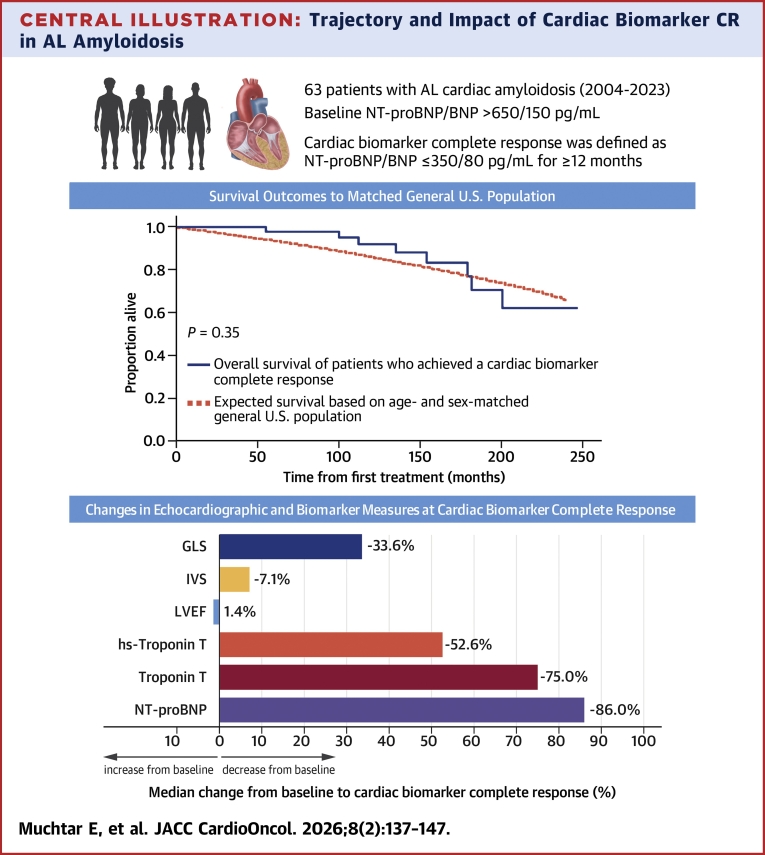
Trajectory and Impact of Cardiac Biomarker CR in AL Amyloidosis Cardiac biomarker complete response (CR), defined as sustained reduction of N-terminal pro–B-type natriuretic peptide (NT-proBNP) or B-type natriuretic peptide (BNP) to ≤350 or 80 pg/mL, signifies deep biochemical cardiac recovery in amyloid light chain (AL) amyloidosis and is associated with long-term survival similar to that of the general population. Despite this favorable prognostic profile, echocardiographic abnormalities consistent with cardiac amyloid involvement may persist. These findings underscore the importance of early diagnosis and timely, effective clone-directed therapy. GLS = global longitudinal strain; hs = high-sensitivity; IVS = interventricular septal thickness; LVEF = left ventricular ejection fraction.

## Methods

After securing Institutional Review Board approval, we screened patients with AL amyloidosis diagnosed between 2004 and 2023 who were evaluated at the Mayo Clinic in Rochester, Minnesota, within 90 days of diagnosis. Eligible patients had cardiac involvement confirmed by imaging (echocardiography or cardiac magnetic resonance imaging) and Congo red–positive tissue from a noncardiac site or cardiac biopsy. Patients also had to be evaluable for cardiac biomarker response, defined by baseline NT-proBNP >650 pg/mL or BNP >150 pg/mL.^9^ Among these, we included patients who achieved a cardiac biomarker CR sustained for at least 12 months.

We use the term *cardiac biomarker CR* to describe this response criterion; it is equivalent to the cardiac CR previously described^9^ but more precisely reflects the biochemical nature of the response. NT-proBNP was available for nearly all patients, and BNP was used in a small subset in which NT-proBNP data were incomplete or unavailable. Heart transplant recipients were excluded. The final cohort comprised 63 patients, including 15 previously reported in a multicenter work^9^ but characterized here in greater detail.

Cardiac staging was determined using the European adaptation of the Mayo 2004 model^7^ with standardized biomarker cutoffs.^10^ For troponin T values less than the laboratory limit of detection (<0.01 ng/mL), a value of 0.005 ng/mL was assigned. Electrocardiograms and echocardiograms were collected at diagnosis and within a 6-month window around the date of cardiac biomarker CR. All electrocardiograms were obtained at the Mayo Clinic. Most echocardiograms were obtained at the Mayo Clinic (84% at diagnostic and 88% at cardiac biomarker CR).

Hematologic response was assessed per consensus criteria^11^ at 3, 6, and 12 months after treatment initiation and at best response before cardiac biomarker CR. Cardiac biomarker response, according to NT-proBNP-graded criteria,^9^ was evaluated at 6, 12, 24, 36, 48, and 60 months following first-line therapy and at the first documented cardiac biomarker CR. To account for variability in visit timing, we used prespecified windows around each assessment: ±1 month at 6 months, ±2 months at 12 months, and ±3 months at 24, 36, 48, and 60 months. When multiple measurements were available within a window, the value closest to the target time point was used. If a landmark cardiac response assessment was unavailable, response was imputed by carrying forward the most recent prior cardiac response, when feasible; this approach was used in 8.2% to 10.7% of patients at designated time points. Additionally, 1, 5, and 12 patients lacked sufficient follow-up for the 36-, 48-, and 60-month landmarks, respectively.

Cardiac progression was defined as any of the following sustained changes: an increase of ≥30% and ≥300 pg/mL in NT-proBNP (or ≥70 pg/mL in BNP) from nadir, an increase of ≥33% in troponin T or high-sensitivity troponin T from nadir, or a decline of ≥10% in left ventricular ejection fraction (LVEF) from the best recorded value.^9^ A temporary increase in NT-proBNP or BNP above the cardiac biomarker CR threshold was defined as an increase that persisted for <12 months and returned to the cardiac biomarker CR threshold within this period, regardless of whether it met the progression criteria.

### Statistical analysis

Descriptive statistics included means, medians, interquartile ranges, and ranges. Categorical variables were compared using the chi-square or Fisher exact test, and nonparametric group comparisons used the Wilcoxon rank sum test. For paired analyses of cardiac parameters, McNemar and Wilcoxon signed rank tests were used. For dichotomized outcomes, we assumed a binomial distribution and calculated proportions with 95% exact binomial CIs.

Follow-up duration was estimated using the reverse Kaplan-Meier method. Kaplan-Meier curves were used for overall survival, calculated from initiation of first-line therapy to death or last follow-up. To compare survival in the cardiac biomarker CR cohort with that of the general U.S. population, we calculated expected survival on the basis of age, biological sex, and treatment year. Observed vs expected hazards were compared using generalized linear models, which is equivalent to a 1-sample log-rank test of whether observed survival matches expected survival in an age-, sex-, and year-matched general U.S. population. *P* values <0.05 were considered to indicate statistical significance. All analyses were performed using JMP version 19.0.0 (SAS Institute) and R version 4.2.2 (R Foundation for Statistical Computing).

## Results

### Baseline characteristics

We screened 2,554 patients with AL amyloidosis seen at our institution during the study period. Of these, 1,754 (68.7%) had cardiac involvement, and 1,342 were evaluable for cardiac biomarker response. Overall, 63 AL amyloidosis patients (4.7%) achieved cardiac biomarker CR. The proportion achieving cardiac biomarker CR was higher in the second period (2014-2023; 6.4% [38 of 591]) than in the first period (2004-2013; 3.3% [25 of 751]). The cohort had a median age of 57 years (Q1-Q3: 49-66 years), and 63.5% were man. Lambda-restricted disease was present in 63.5% of patients. The median difference between involved and uninvolved free light chains (dFLC) was 429 mg/L (Q1-Q3: 143-708 mg/L). Extracardiac involvement was observed in 63% of cardiac patients, including renal (52.4%), liver (15.9%), autonomic nerve (14.3%), luminal gastrointestinal (11.1%), and peripheral nerve (9.5%) involvement. Compared to patients with cardiac involvement from the same study period who did not achieve cardiac biomarker CR, those who achieved cardiac biomarker CR were younger and had a higher proportion of kappa isotype, higher estimated glomerular filtration rates (eGFRs), lower cardiac biomarker levels, and lower cardiac stage at diagnosis (Table 1).

**Table 1:** Baseline Characteristics of Cardiac Biomarker CR vs Non–Cardiac Biomarker CR Patients in the Study Period

	Cardiac Biomarker CR (n = 63)	Non–Cardiac Biomarker CR (n = 1,279)	*P* Value
Age, y	57 (49-66)	65 (59-72)	<0.001
Male	63.5	63.3	0.96
Lambda isotype	63.5	75.6	0.03
Baseline dFLC, mg/L	429 (143-708)	308 (132-734)	0.22
BMPCs, %	10 (7-20)	10 (6-20)	0.53
eGFR, mL/min/1.73 m^2^	73 (57-91)	59 (41-76)	<0.001
NT-proBNP, pg/mL	1,977 (1,393-3,026) (n = 62)	4,326 (2,048-9,344) (n = 1,156)	<0.001
BNP, pg/mL	297 (242-617) (n = 13)	731 (380-1,340) (n = 492)	0.018
Troponin T, ng/mL	0.02 (<0.01-0.05) (n = 43)	0.05 (0.02-0.11) (n = 924)	0.003
High-sensitivity troponin T, ng/L	49 (38-79) (n = 19)	73 (40-132) (n = 257)	0.055
Troponin I, ng/mL	0.025 (0.003-0.12) (n = 10)	0.06 (0.02-0.18) (n = 124)	0.101
Cardiac stage			<0.001
I	0	0	
II	57.1	39.4	
IIIA	36.5	31.4	
IIIB	6.4	29.2	

Values are median (Q1-Q3) or %. Cardiac biomarkers (natriuretic peptides and troponins) may be reported from more than one assay per patient.

BMPC = bone marrow plasma cell; BNP = B-type natriuretic peptide; CR = complete response; dFLC = difference between involved and uninvolved light chains; eGFR = estimated glomerular filtration rate; NT-proBNP = N-terminal pro–B-type natriuretic peptide.

Table 2 presents cardiac parameters at diagnosis. The median NT-proBNP concentration was 1,977 pg/mL (Q1-Q3: 1,393-3,026 pg/mL), the median troponin T concentration was 0.02 ng/mL (Q1-Q3: <0.01-0.05 ng/mL), and the median high-sensitivity troponin T concentration was 49 ng/L (Q1-Q3: 38-79 ng/L). Cardiac stage was II in 57.1%, IIIA in 36.5%, and IIIB in 6.4%. Key echocardiographic findings included a median LVEF of 63% (Q1-Q3: 58%-68%), interventricular septal thickness of 14 mm (Q1-Q3: 12-16 mm), and average longitudinal left ventricular strain of −12% (Q1-Q3: −15% to −10%). Among 50 baseline electrocardiograms, 96.0% showed sinus rhythm.

**Table 2:** Cardiac Assessment at Diagnosis and at the Time of Cardiac Biomarker CR

	n	At Baseline	n	At Cardiac Biomarker CR	Median Percentage Difference (Q1-Q3)	*P* Value
Cardiac biomarkers						
NT-proBNP, pg/mL	62	1,977 (1,393-3,026)	62	265 (220-307)	−86.9 (−93.0 to −80.6)	<0.001
BNP, pg/mL	13	297 (242-617)	2	41, 68	—	—
Troponin T, ng/mL	43	0.02 (<0.01-0.05)	36	<0.01 (<0.01-0.01)	−75.0 (−87.5 to 0)	<0.001
High-sensitivity troponin T, ng/L	19	49 (38-79)	23	26 (20-33)	−52.6 (−61.3 to −30.1)	<0.001
Troponin I, ng/mL	10	0.025 (0.003-0.12)	—	—	—	—
Cardiac stage I/II/IIIA/IIIB, %	62		59		—	<0.001
I		0		86		
II		57.1		14		
IIIA		36.5		0		
IIIB		6.4		0		
eGFR, mL/min/1.73 m^2^	63	73 (57-91)	59	66 (56-79)	−6.8 (−21.0 to +11.4)	0.11
Echocardiography						
LVEF, %	62	63.5 (58-68)	57	64 (61-68)	+1.4 (−5.9 to +8.5)	0.19
LVEF ≥55%		83.9		96.5	—	0.010
IVS thickness, mm	61	14 (12-16)	55	13 (11-15)	−7.1 (−20 to +1.7)	<0.001
IVS thickness ≤12 mm		26.2		43.6	—	0.02
LV average longitudinal strain, %	54	−12 (−10 to −15)	54	−17 (−14 to −18)	−33.6 (−51.1 to −8.2)	<0.001
LV average longitudinal strain ≤−18%		3.7		27.8	—	0.001
Cardiac index, L/min/m^2^	49	2.9 (2.6-3.6)	51	3.0 (2.7-3.5)	+2.9 (−13.4 to +25.0)	0.32
Cardiac index ≥2.5 L/min/m^2^		79.6		84.3	—	0.47
Stroke volume index, mL/m^2^/beat	50	38 (33-46)	51	44 (39-49)	+17.8 (+2.4 to +35.8)	<0.001
Stroke volume index, ≥35 mL/m^2^/beat		66		90.2		0.007
E/A ratio	45	1.4 (0.8-2.0)	54	1 (0.7-1.5)	−23.2 (−42.0 to +12.0)	0.002
E/A ratio <2		73.3		88.9	—	0.002
Mitral E/e′ ratio	49	16.7 (14-20)	53	15 (10-17.5)	−18.3 (−35.7 to +10.6)	0.01
Mitral E/e′ ratio <15		30.6		47.1		0.13
RV systolic pressure, mm Hg	38	35 (28-44)	31	29 (26-32)	−10.8 (−34.1 to +3.6)	0.03
RV systolic pressure <35 mm Hg		50		83.9	—	0.01
ECG rhythm	50		40		—	
Sinus		96.0		92.5		
Atrial fibrillation/flutter		4.0				
Pacemaker		2		7.5		
Wandering atrial pacemaker		2				

Values are median (Q1-Q3) or %.

ECG = electrocardiographic; IVS = interventricular septal; LV = left ventricular; LVEF = left ventricular ejection fraction; RV = right ventricular; other abbreviations as in Table 1.

### Treatment and hematological response

First-line treatment was autologous stem cell transplantation (ASCT) in 54.0% of patients. Other first-line regimens included proteasome inhibitor–containing therapy (primarily cyclophosphamide, bortezomib, and dexamethasone [22.2%]), daratumumab-containing therapy (mostly daratumumab, cyclophosphamide, bortezomib, and dexamethasone [12.7%]), melphalan-dexamethasone (9.5%), and lenalidomide (1.6%). The use of ASCT decreased from 64.0% (95% CI: 42.5%-82.0%) in 2004-2013 to 47.4% (95% CI: 31.0%-64.2%) in 2014-2023. Among ASCT recipients, 70.6% received induction therapy, 85.3% received full-dose melphalan conditioning, and 26.5% received post-ASCT maintenance therapy. Therapies by period are provided in Supplemental Table 1.

Hematological CR rates were 32.2%, 49.1%, and 63.3% at 3, 6, and 12 months, respectively. Rates of very good partial response or better were 71%, 88%, and 97% at the same time points. Before cardiac biomarker CR, the best hematological response was CR in 76.2% of patients, very good partial response in 20.6%, and partial response in 3.2%. The median time to best hematological response was 4.3 months (Q1-Q3: 2.6-8.0 months).

The median dFLC was 16 mg/L (Q1-Q3: 4.2-40 mg/L) at 3 months, 7.6 mg/L (Q1-Q3: −0.2 to 18.1 mg/L) at 6 months, 1.6 mg/L (Q1-Q3: −3.1 to 8.2 mg/L) at 12 months, and 4.8 mg/L (Q1-Q3: 0.1-10.5 mg/L) at best response before cardiac biomarker CR, representing a 96% to 99% reduction from baseline.

### Achievement of cardiac biomarker CR

The median time from treatment initiation to first documented cardiac biomarker CR was 20.6 months (Q1-Q3: 11.8-27.3 months). At cardiac biomarker CR, the median NT-proBNP concentration was 265 pg/mL (Q1-Q3: 220-307 pg/mL) in 62 patients; 2 patients had BNP concentrations of 68 and 41 pg/mL at cardiac biomarker CR. Median troponin T (n = 36), high-sensitivity troponin T (n = 23), and eGFR (n = 59) at cardiac biomarker CR were <0.01 ng/mL, 26 ng/L, and 66 mL/min/1.73 m^2^, respectively.

At cardiac biomarker CR, 41% of patients were receiving diuretic medications, including 33% receiving loop diuretic agents. The median daily furosemide-equivalent dose was 0.5 mg/kg (Q1-Q3: 0.3-1.0 mg/kg). NT-proBNP levels at cardiac biomarker CR did not differ by loop diuretic use (*P* = 0.62).

### Changes in cardiac biomarkers and echocardiographic findings at cardiac biomarker CR

Table 2 summarizes changes in cardiac parameters between diagnosis and cardiac biomarker CR. Median reductions in NT-proBNP, troponin T, and high-sensitivity troponin T concentrations were 86.9%, 75%, and 52.6%, respectively (*P* ≤ 0.001 for all), and cardiac stage I predominated at cardiac biomarker CR (86%). Echocardiographic parameters also improved significantly, with average left ventricular longitudinal strain showing the greatest median improvement from baseline (33.6%; *P* < 0.001). Other notable median changes included E/A ratio (−23.2%), mitral E/e′ ratio (−18.3%), stroke volume index (+17.8%), and interventricular septum (−7.1%). LVEF and cardiac index did not change significantly; however, the proportion of patients with LVEFs ≥55% increased from 83.9% to 96.5% (*P* = 0.010). Among 60 patients with follow-up echocardiography, 38 (63.3%) achieved strain of −18% or more negative, with a median time of 43.4 months (Q1-Q3: 21-53 months).

### Longitudinal assessment of cardiac response

**Figure 1:**
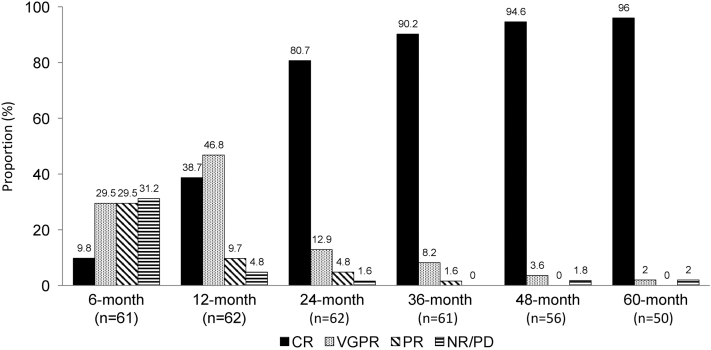
Cardiac Biomarker Response at Landmarks (6, 12, 24, 36, 48, and 60 Months After Treatment Initiation) This figure shows the longitudinal cardiac biomarker response after treatment in patients with amyloid light chain (AL) cardiac amyloidosis. Cardiac biomarker complete response (CR) is defined as N-terminal pro–B-type natriuretic peptide (NT-proBNP) or B-type natriuretic peptide (BNP) ≤350 or 80 pg/mL. The proportion of patients achieving cardiac biomarker CR increased over time, reaching 96% at 60 months. Other response categories include very good partial response (VGPR; (>60% reduction from baseline without meeting CR), partial response (PR; >30% to ≤60% reduction from baseline), and no response (NR; ≤30% reduction from baseline), defined by percentage reductions in NT-proBNP or BNP from baseline. This figure highlights the delayed but progressive nature of cardiac biomarker recovery in AL cardiac amyloidosis. PD = progressive disease (>30% and >300 pg/mL [for BNP >30% and >70 pg/ mL] increase from nadir not precipitated by infection, elevated creatinine, or cardiac arrhythmia).

Figure 1 and Table 3 detail longitudinal cardiac biomarker response. The proportion of patients achieving cardiac biomarker CR increased over time: 90% by 36 months, 95% by 48 months, and 96% by 60 months. The median duration of cardiac biomarker CR was 16.8 years (95% CI: 10.6 years to not reached).

**Table 3:** Cardiac Biomarker Levels and eGFR at Landmark Time Points

	6 mo	12 mo	24 mo	36 mo	48 mo	60 mo
NT-proBNP, pg/mL	810 (494-1,550)	413 (274-635)	229 (163-317)	245 (159-298)	208 (159-298)	185 (135-250)
Troponin T, ng/mL	0.01 (<0.01-0.03)	<0.01 (<0.01-0.018)	<0.01 (<0.01-0.01)	<0.01 (<0.01-0.01)	<0.01 (<0.01-<0.01)	<0.01 (<0.01-0.01)
High-sensitivity troponin T, ng/L	40 (30-53)	28 (22-48)	24 (16-31)	19 (14-26)	15 (11-21)	20 (12-25)
eGFR, mL/min/1.73 m^2^	66 (56-81)	68 (56-82)	69 (60-80)	67 (59-79)	66 (57-79)	68 (53-79)

Values are median (Q1-Q3).

NT-proBNP values at 6, 12, 24, 36, 48, and 60 months were available for 54, 55, 56, 53, 49 and 43 patients. Troponin values at 6, 12, 24, 36, 48, and 60 months were available for 31, 32, 28, 26, 21, and 18 patients. High-sensitivity troponin T values at 6, 12, 24, 36, 48 and 60 months were available for 17, 19, 21, 20, 22, and 21 patients. eGFR values at 6, 12, 24, 36, 48, and 60 months were available for 55, 52, 54, 52, 47 and 43 patients.

Abbreviations as in Table 1.

During follow-up, 46% of patients had temporary increases in NT-proBNP (or BNP) above the cardiac biomarker CR threshold that subsequently returned to the cardiac biomarker CR range. NT-proBNP levels at cardiac biomarker CR did not differ between patients with and those without temporary increases (*P* = 0.59); however, patients without temporary increases had lower nadir NT-proBNP concentrations (median 110 vs 162 pg/mL in those with temporary increases; *P* < 0.001). A temporary increase in NT-proBNP or BNP was not associated with subsequent cardiac progression (*P* = 0.60).

### Hematological progression

Twenty-eight patients (44.4%) required additional clone-directed therapy, initiated a median of 4.6 years after initial treatment (Q1-Q3: 2.2-7.2 years). Hematologic progression occurred in 19.0% per formal criteria, and 25.4% initiated subsequent therapy after an increase in involved free light chains that did not meet hematologic progression criteria. At the initiation of subsequent therapy, the median dFLC was 58 mg/L (Q1-Q3: 27-102 mg/L), corresponding to 13% of baseline (Q1-Q3: 6%-30%). Of these 28 patients, 9 received 1 additional line, 9 received 2 additional lines, and 10 received more than 2 additional lines.

### Cardiac progression

NT-proBNP progression was observed in 9 patients (14%). This progression occurred a median of 7.6 years from initial cardiac biomarker CR (Q1-Q3: 3.5-10.2 years), with a median NT-proBNP concentration of 784 pg/mL (Q1-Q3: 580-2,300 pg/mL) at progression. Of these 9 patients, 7 also had troponin progression, and 1 had LVEF progression.

NT-proBNP progression was associated with the initiation of new clone-directed therapy (n = 3; immunomodulatory-based regimens in 2 patients and an alkylator plus dexamethasone in 1 patient), worsening renal function (n = 2, including 1 who also initiated new clone-directed therapy), new-onset atrial fibrillation (n = 2), and severe tricuspid regurgitation (n = 1).

### Survival

The median follow-up duration was 9.3 years from treatment initiation (95% CI: 7.8-12.3 years). During follow-up, 8 patients (4 men, 4 women; 12.7%) died. Five- and 10-year overall survival rates were 98.0% (95% CI: 94.3%-100%) and 92.1% (95% CI: 83.8%-100%), respectively. Among the 8 patients who died, the median survival time was 12.1 years (range: 4.6-16.7 years), and the median age at death was 76.8 years (range: 60.7-88.8 years).

Two deaths were unrelated to AL amyloidosis: Alzheimer’s disease in 1 patient and intracranial hemorrhage in 1 patient receiving anticoagulation for prior pulmonary embolism. Two patients died suddenly, possibly related to AL amyloidosis, at 81 and 88 years of age; both were in remission and had not received active treatment for more than 10 years. The remaining 4 deaths were attributed to amyloidosis-related causes (n = 1 each): septic shock while receiving daratumumab and immunosuppressants after kidney transplantation, hematologic and cardiac progression, ischemic bowel during daratumumab treatment (while in remission), and chronic obstructive pulmonary disease exacerbation while receiving clone-directed therapy (venetoclax).

**Figure 2:**
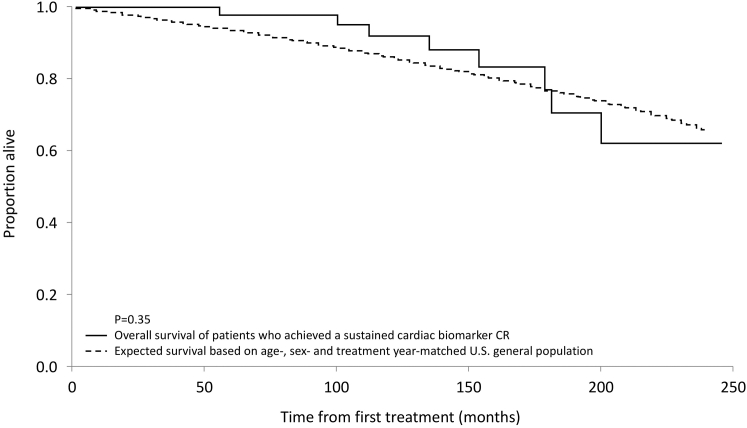
Survival of Patients With AL Amyloidosis Achieving Cardiac Biomarker CR Compared With a Matched General U.S. Population The figure compares observed overall survival in patients with AL amyloidosis who achieved sustained cardiac biomarker CR with expected survival in an age-, sex-, and treatment year–matched general U.S. population. The Kaplan-Meier curve shows that survival in the cardiac biomarker CR cohort is similar to that of the general population. Five- and 10-year overall survival rates were 98.0% and 92.1%, respectively, supporting an association between cardiac biomarker CR and excellent long-term survival. Abbreviations as in Figure 1.

Survival among patients who achieved cardiac biomarker CR was compared with that of a matched general U.S. population on the basis of age, biological sex, and year of treatment initiation. As shown in Figure 2, observed survival in the cardiac biomarker CR cohort was similar to expected survival in the matched general population (*P* = 0.35).

## Discussion

This study provides the first comprehensive characterization of patients with AL amyloidosis who achieve cardiac biomarker CR. Although cardiac biomarker CR is associated with optimal cardiac recovery and excellent long-term survival comparable with the general population, it is important to recognize that this “complete” response reflects biochemical and prognostic improvement rather than complete restoration of cardiac structure.

In our cohort, echocardiographic parameters relevant to AL cardiac amyloidosis (CA) improved but did not fully normalize in all patients (Central Illustration). High-sensitivity troponin T levels also decreased substantially from baseline but often remained above the normal range, suggesting persistent subclinical myocardial stress or injury. Together, these findings indicate that despite cardiac biomarker CR, some residual cardiac abnormalities may persist.

A misconception persists that therapy for AL amyloidosis is relatively ineffective. In contrast, our data highlight a subgroup that achieves excellent outcomes with clone-directed therapy, including substantial cardiac functional recovery.

Most patients in this study presented with early-stage cardiac involvement (cardiac stage II in 59%), and only 4 had cardiac stage IIIB. In comparison, patients from the same study who did not achieve cardiac biomarker CR had more advanced cardiac stage, underscoring the importance of early diagnosis.

Accordingly, in patients with thickened ventricular walls, heart failure symptoms and preserved LVEF, measurement of immunoglobulin light chains should be incorporated into the diagnostic evaluation to facilitate earlier recognition of AL CA and increase the likelihood of a favorable outcome.

Achieving durable cardiac biomarker CR remains challenging. Over 20 years at our referral center, only 63 patients met this criterion, representing 4.7% of cardiac-evaluable patients. However, rates of cardiac biomarker CR appear to be increasing with more effective therapies,^12^^,^^13^ and this trend may continue.

A prior multicenter study reported cardiac biomarker CR in as many as 16% of patients as their best response,^9^ but studies of cardiac biomarker response can be affected by selection bias. Fitter patients are more likely to survive long enough to be assessed, whereas patients who die early are underrepresented. Several additional factors may also contribute to the low rate observed in our cohort.

First, advanced organ dysfunction reduces the likelihood of complete or near complete reversal of damage.^9^^,^^14^ Because many patients present with advanced cardiac involvement, fewer achieve cardiac biomarker CR. Second, structural consequences of CA (eg, valvular regurgitation, atrial enlargement) may predispose patients to volume overload and atrial fibrillation, which can increase NT-proBNP beyond what might be expected from myocardial involvement alone.^15^ Third, kidney involvement is common in AL amyloidosis, and reduced renal function can further increase NT-proBNP. In our study, patients who achieved cardiac biomarker CR had relatively preserved renal function, which may have reduced natriuretic peptide elevations related to reduced eGFR.^16^ Finally, we required cardiac biomarker CR to persist for more than 12 months to account for natural fluctuations in NT-proBNP, which reduced the number of eligible patients.

Natriuretic peptides are commonly used to assess cardiac biomarker response in AL CA, but levels can fluctuate. In our cohort, nearly one-half of patients had at least 1 NT-proBNP or BNP measurement above the cardiac biomarker CR threshold after achieving cardiac biomarker CR, particularly when the nadir NT-proBNP or BNP was closer to the threshold. This is expected given the many factors that influence natriuretic peptide levels.^17^ Therefore, transient increases should be interpreted cautiously in clinical decision-making and may not necessarily reflect loss of treatment response. Potential contributors include valvular regurgitation, fluid retention (possibly due to dietary changes), medication effects or nonadherence to medication, atrial fibrillation, and reduced renal clearance.

Cardiac progression was uncommon, affecting approximately 1 in 7 patients; however, the need for subsequent clone-directed therapy remained substantial, with nearly one-half requiring additional treatment during follow-up. Thus, although cardiac biomarker CR appears to confer some protection against cardiac progression, it does not eliminate the risk for hematologic relapse, even among patients who achieved hematologic CR with initial therapy.

Early intervention strategies^18^ may have contributed to the low rate of cardiac progression, supported by lower dFLCs at initiation of subsequent therapy compared with baseline. These findings highlight the need for lifelong monitoring to enable timely therapeutic intervention and reduce the risk for irreversible organ deterioration, particularly because cardiac progression remains a strong negative prognostic factor in AL amyloidosis.^19^ Four of the 8 deaths occurred while patients were receiving therapy, which may reflect underlying disease biology rather than treatment-related mortality. However, at least 2 deaths were infection related, a recognized risk of clone-directed treatments, underscoring the importance of vigilant infection monitoring and timely intervention during therapy.

Several baseline characteristics differed between patients who achieved cardiac biomarker CR and those who did not. Patients who achieved cardiac biomarker CR were, on average, 8 years younger than those who did not, whose median age was similar to that of our overall referral population.^20^ This age difference likely reflects the higher proportion of patients who underwent ASCT, as ASCT candidates are generally younger and fitter.^21^ Younger age may also be associated with greater tissue repair capacity, potentially increasing the likelihood of achieving cardiac biomarker CR.

We did not observe a distinct pattern of extracardiac organ involvement compared with prior reports from our center.^22^ This suggests that extracardiac involvement is not necessarily a barrier to achieving cardiac biomarker CR, although, as noted earlier, patients who achieved cardiac biomarker CR typically had preserved eGFR. Consistent with our prior work on predictors of cardiac biomarker response, we observed a higher frequency of kappa-restricted disease (although lambda-restricted disease remained more common overall) and higher baseline dFLCs.^23^ These findings raise the possibility that clonal characteristics influence the depth and kinetics of cardiac biomarker response. Patients with greater light chain–mediated toxicity (rather than predominantly irreversible amyloid deposition)^24^ may have a greater potential for organ recovery once circulating light chains are eliminated. These observations warrant validation in independent cohorts.

### Study limitations

Limitations include the single-center, retrospective design and the lack of a comparator group. We acknowledge that defining cardiac biomarker CR using fixed NT-proBNP and BNP thresholds is inherently arbitrary and dichotomizes a continuous variable; as such, it may not reflect a biological inflection point or complete normalization of cardiac function. Assessment of cardiac recovery was also limited by the absence of serial imaging and functional evaluations such as cardiac magnetic resonance imaging, 6-minute walk testing, and cardiopulmonary exercise testing. Incorporating these measures could better define the extent of cardiac recovery in patients who achieve cardiac biomarker CR according to natriuretic peptides.

## Conclusions

This study provides the first detailed analysis of patients with AL amyloidosis achieving cardiac biomarker CR. Cardiac biomarker CR was associated with excellent long-term outcomes, including improved survival and a relatively low risk for cardiac progression.

### Data sharing statement

The dataset used in this study is not publicly available, because of the sensitive nature of electronic health records and the need to protect patient anonymity. An anonymized dataset may be made available upon reasonable request to the corresponding author.

Perspectives **COMPETENCY IN MEDICAL KNOWLEDGE:** Cardiac biomarker CR, defined as sustained reduction of NT-proBNP or BNP to ≤350 or 80 pg/mL, is a novel marker of cardiac recovery in AL CA. In this study, patients who achieved cardiac biomarker CR had sustained improvements in cardiac biomarkers and cardiac function and long-term survival similar to that of the general population. These findings support early, effective clone-directed therapy to maximize the likelihood of achieving deep cardiac biomarker response, which is associated with favorable long-term outcomes in AL CA.

**TRANSLATIONAL OUTLOOK:** Future research is needed to define the mechanisms underlying the high long-term survival observed in patients who achieve cardiac biomarker CR, particularly because echocardiographic parameters may not fully normalize. Priorities include 1) identifying optimal chemotherapeutic regimens and novel agents that increase the rate of cardiac biomarker CR; 2) developing and validating imaging or molecular markers that predict cardiac biomarker CR earlier than NT-proBNP or BNP or detect residual cardiac tissue damage despite biomarker normalization; and 3) determining whether sustained cardiac biomarker CR supports the de-escalation or cessation of therapy to reduce treatment-related toxicity. These directions underscore the clinical value of biomarker-guided management and will inform future strategies to achieve deep cardiac remission more consistently in AL CA.

## Funding Support and Author Disclosures

Dr Muchtar has received consulting fees (paid to the institution) from Protego. Dr Dispenzieri has received research funding from Janssen, Alexion, Takeda, HaemaloiX, Alnylam, Bristol Myers Squibb, and Pfizer; and is a consultant for Alexion, Takeda, and Bristol Myers Squibb. Dr Kapoor has received research funding from Loxo Pharmaceuticals, Karyopharm, GlaxoSmithKline, AbbVie, Sanofi, BeiGene, Ichnos, Bristol Myers Squibb, Regeneron, and Amgen; is a board of directors or advisory committee member for Kite, Oncopeptides, GlaxoSmithKline, Pharmacyclics, AbbVie, Janssen, Mustang Bio, Sanofi, BeiGene, Angitia Bio, and X4 Pharmaceuticals; and is a consultant for CVS Caremark and Keosys. Dr Dingli is a consultant for Genentech, Sorrento, Janssen, Novartis, Regeneron, Bristol Myers Squibb, Sanofi, Merck Sharpe & Dohme, Apellis, and Alexion; has received honoraria from Sorrento, Janssen, Novartis, Regeneron, Bristol Myers Squibb, Sanofi, Merck Sharpe & Dohme, Apellis, and Alexion; and has received research funding from K36 Therapeutics and Apellis. Dr Kourelis has received research funding from Novartis and Pfizer. Dr Leung holds stock options in Checkpoint Therapeutics and AbbVie. Dr Kumar is a board of directors or advisory committee member for Celgene, Takeda, Kite, MedImmune/AstraZeneca, Adaptive, AbbVie, Janssen; has received research funding from Celgene, Roche, Merck, Takeda, Kite, MedImmune/AstraZeneca, Adaptive, AbbVie, Janssen, Novartis, and Sanofi; and has participated on an independent review committee for Oncopeptides. Dr Gertz has served as a consultant for Millennium Pharmaceuticals; and has received honoraria from Celgene, Millennium Pharmaceuticals, Onyx Pharmaceuticals, Novartis, GlaxoSmithKline, Prothena, Ionis Pharmaceuticals, and Amgen. All other authors have reported that they have no relationships relevant to the contents of this paper to disclose.

## References

[1] Sanchorawala V. (2024). Systemic light chain amyloidosis. N Engl J Med.

[2] Grogan M., Dispenzieri A. (2015). Natural history and therapy of AL cardiac amyloidosis. Review. Heart Fail Rev.

[3] Gertz M.A., Dispenzieri A. (2020). Systemic amyloidosis recognition, prognosis, and therapy: a systematic review. JAMA.

[4] Gustine J.N., Staron A., Mendelson L. (2023). Predictors of treatment response and survival outcomes in patients with advanced cardiac AL amyloidosis. Blood Adv.

[5] Dispenzieri A., Gertz M.A., Kyle R.A. (2004). Serum cardiac troponins and N-terminal pro-brain natriuretic peptide: a staging system for primary systemic amyloidosis. J Clin Oncol.

[6] Kumar S., Dispenzieri A., Lacy M.Q. (2012). Revised prognostic staging system for light chain amyloidosis incorporating cardiac biomarkers and serum free light chain measurements. J Clin Oncol.

[7] Palladini G., Sachchithanantham S., Milani P. (2015). A European collaborative study of cyclophosphamide, bortezomib, and dexamethasone in upfront treatment of systemic AL amyloidosis. Blood.

[8] Muchtar E., Geyer S., Merlini G., Gertz M.A. (2024). Patients with a cardiac complete response in AL amyloidosis have survival rates similar to those of a matched general population. Blood.

[9] Muchtar E., Dispenzieri A., Wisniowski B. (2023). Graded cardiac response criteria for patients with systemic light chain amyloidosis. J Clin Oncol.

[10] Muchtar E., Kumar S.K., Gertz M.A. (2019). Staging systems use for risk stratification of systemic amyloidosis in the era of high-sensitivity troponin T assay. Blood.

[11] Palladini G., Dispenzieri A., Gertz M.A. (2012). New criteria for response to treatment in immunoglobulin light chain amyloidosis based on free light chain measurement and cardiac biomarkers: impact on survival outcomes. J Clin Oncol.

[12] Kastritis E., Leleu X., Arnulf B. (2020). Bortezomib, melphalan, and dexamethasone for light-chain amyloidosis. J Clin Oncol.

[13] Kastritis E., Palladini G., Minnema M.C. (2021). Daratumumab-based treatment for immunoglobulin light-chain amyloidosis. N Engl J Med.

[14] Muchtar E., Wisniowski B., Geyer S. (2024). Graded organ response and progression criteria for kidney immunoglobulin light chain amyloidosis. JAMA Oncol.

[15] Tomasoni D., Aimo A., Porcari A. (2024). Prevalence and clinical outcomes of isolated or combined moderate to severe mitral and tricuspid regurgitation in patients with cardiac amyloidosis. Eur Heart J Cardiovasc Imaging.

[16] Santos-Araujo C., Leite-Moreira A., Pestana M. (2015). Clinical value of natriuretic peptides in chronic kidney disease. Nefrologia.

[17] de Lemos J.A., McGuire D.K., Drazner M.H. (2003). B-type natriuretic peptide in cardiovascular disease. Lancet.

[18] Milani P., Gertz M.A., Merlini G., Dispenzieri A. (2017). Attitudes about when and how to treat patients with AL amyloidosis: an international survey. Amyloid.

[19] Palladini G., Milani P., Foli A. (2018). Presentation and outcome with second-line treatment in AL amyloidosis previously sensitive to nontransplant therapies. Blood.

[20] Muchtar E., Gertz M.A., Kumar S.K. (2017). Improved outcomes for newly diagnosed AL amyloidosis between 2000 and 2014: cracking the glass ceiling of early death. Blood.

[21] Muchtar E., Dispenzieri A., Sanchorawala V. (2025). A model for predicting day-100 stem cell transplant-related mortality in AL amyloidosis. Bone Marrow Transplant.

[22] Muchtar E., Gertz M.A., Kyle R.A. (2019). A modern primer on light chain amyloidosis in 592 patients with mass spectrometry-verified typing. Mayo Clin Proc.

[23] Rees M.J., Geyer S., Yohannan B. (2025). Clonal plasma cell features in light-chain amyloidosis are associated with depth and timing of cardiac response independent of hematologic response. Haematologica.

[24] Merlini G., Comenzo R.L., Seldin D.C., Wechalekar A., Gertz M.A. (2014). Immunoglobulin light chain amyloidosis. Expert Rev Hematol.

