# Supplementary material for: Cardiac Biomarker Complete Response in AL Amyloidosis: Characteristics, Cardiac Recovery, and Survival of 63 Patients
Source: JACC CardioOncol. 2026 Apr 21;8(2):137–47. doi: 10.1016/j.jaccao.2026.01.004 (PMC13133647; doi:10.1016/j.jaccao.2026.01.004)
Supplement: Supplementary Table 1 [file mmc1.docx]

**Supplemental material to:**

Cardiac biomarker Complete Response in AL Amyloidosis: Characteristics, Recovery, and Survival of 63 Patients. Muchtar E, Geyer S, Dispenzieri A, et al

| **Supplementary Table 1.** First-line treatment groups by study periods | | |
| --- | --- | --- |
|  | **2004-2013 period (n=25)** | **2014-2023 period (n=38)** |
| Autologous stem cell transplantation  With induction | 16 (64.0%)  9 (36.0%) | 18 (47.4%)  15 (39.5%) |
| Bortezomib-based | 3 (12.0%) | 11 (28.9%) |
| Daratumumab-based | 0 | 8 (21.1%) |
| Melphalan-dexamethasone | 5 (20.0%) | 1 (2.6%) |
| Lenalidomide alone | 1 (4.0%) | 0 |
